# Impact of Established and Emerging Software Tools on the Metabolite Identification Landscape

**DOI:** 10.3389/ftox.2022.932445

**Published:** 2022-06-21

**Authors:** Anne Marie E. Smith, Kiril Lanevskij, Andrius Sazonovas, Jesse Harris

**Affiliations:** ACD/Labs, Toronto, ON, Canada

**Keywords:** metabolite identification, machine learning, computational chemistry, metabolite prediction, analytical data management

## Abstract

Scientists’ ability to detect drug-related metabolites at trace concentrations has improved over recent decades. High-resolution instruments enable collection of large amounts of raw experimental data. In fact, the quantity of data produced has become a challenge due to effort required to convert raw data into useful insights. Various cheminformatics tools have been developed to address these metabolite identification challenges. This article describes the current state of these tools. They can be split into two categories: Pre-experimental metabolite generation and post-experimental data analysis. The former can be subdivided into rule-based, machine learning-based, and docking-based approaches. Post-experimental tools help scientists automatically perform chromatographic deconvolution of LC/MS data and identify metabolites. They can use pre-experimental predictions to improve metabolite identification, but they are not limited to these predictions: unexpected metabolites can also be discovered through fractional mass filtering. In addition to a review of available software tools, we present a description of pre-experimental and post-experimental metabolite structure generation using MetaSense. These software tools improve upon manual techniques, increasing scientist productivity and enabling efficient handling of large datasets. However, the trend of increasingly large datasets and highly data-driven workflows requires a more sophisticated informatics transition in metabolite identification labs. Experimental work has traditionally been separated from the information technology tools that handle our data. We argue that these IT tools can help scientists draw connections *via* data visualizations and preserve and share results *via* searchable centralized databases. In addition, data marshalling and homogenization techniques enable future data mining and machine learning.

## 1 Introduction

Drug metabolism influences the pharmacokinetics and pharmacodynamics of drug molecules while altering their pharmacological activity and toxicity (Kirchmair et al., 2015; Manikandan and Nagini, 2018). Determining drug metabolism in drug research and development is essential for producing safe and effective medication. The ability to detect metabolites at trace concentrations has dramatically improved recently due to advances in instrumentation such as high-resolution mass spectrometry (HRMS) (Zhu et al., 2011).

However, this raw data does not directly contribute to drug development. Metabolite structures must be elucidated and processed data recorded in a manner that is human-readable and readily shareable/searchable.

Advances in instrumentation have led to challenges with raw data handling. Traditional metabolism identification (MetID) tools, such as manual expert systems, are no longer sufficient to meet the needs of an increasingly complex cheminformatics landscape (Cuyckens, 2018; Géhin and Holman, 2021). In addition, MetID scientists may be part of structure elucidation groups, meaning the software requirements are often subject to competing considerations.

Metabolite prediction tools may use different computational approaches, but they are all limited by the quality and quantity of data available. Therefore, the effectiveness of a MetID team is a function of their software tools, experimental equipment, and data management strategy. This article summarizes the current state of MetID software, including an overview of available commercial applications and perspectives on future innovation.

## 2 Overview of Software-based Techniques

MetID software is used for two main activities, though there is overlap, and many programs do both:

1. Pre-experimental generation of metabolites from a structure
2. Post-experimental analysis of data

Several MetID software packages are commercially available that substantially improve manual techniques in both efficiency and accuracy (Kirchmair et al., 2012; Kazmi et al., 2019).

While these software tools have unique features, most applications have a limited ability to predict metabolites for non-mammals. Processed metabolic data is biased towards mammals, meaning the software cannot reliably predict metabolites produced by plants, insects, or bacteria. This has implications for pesticide development, and environmental toxicology (Hoagland et al., 2000; Hatzios et al., 2001). Research organizations can build in-house biotransformation databases supported by expert knowledge to improve the performance of MetID software for specialized areas.

### 2.1 Pre-Experimental Metabolite Generation

Pre-experimental metabolite generation tools predict metabolites *de novo* based on structure. The three most common approaches for pre-experimental prediction are rule-based, machine learning-based, and docking-based.

These computational strategies are not mutually exclusive, as they deal with different aspects of metabolite prediction. Only rule-based methods generate the structures of potential metabolites. Other techniques estimate the preferred site of metabolism (SoM). Many software packages use multiple approaches to cover the entire metabolite prediction workflow.

#### 2.1.1 Rule-Based Pre-Experimental Metabolite Generation

As the name implies, rule-based prediction software uses empirically-derived rules to predict biotransformations for a given molecule. This software finds possible metabolites by comparing the molecule against an experimental database of metabolic reactions. Alternatively, the algorithm can identify substructures that fulfill the SoM criteria for different reaction types, then assess which transformation(s) will occur. This process is repeated to predict next-generation metabolites.

Rule-based systems offer the advantage of predictions that can be rationally compared to experimentally observed results. Researchers can assess how specific metabolites were predicted, allowing experts to apply their knowledge. Since these tools are limited by the set of rules available, software updates and in-house data are needed to ameliorate this constraint. This can be a time-consuming process.

Examples of rule-based MetID software include

• Nexus Meteor: A knowledge-based system that uses a biotransformation dictionary expressed as generic reaction descriptions. These biotransformations are applied to structures using reasoning rules (Marchant et al., 2008).

• BioTransformer: Hybrid software tool that predicts xenobiotic metabolism in several systems (Djoumbou-Feunang et al., 2019). It uses a biotransformation database (MetXBioDB), a reaction knowledgebase, and a reasoning engine that incorporates machine learning algorithms, such as CypReact (Tian et al., 2018) to predict enzyme selectivity.

• GLORYx: Phase I and II metabolite prediction software. GLORYx employs a hybrid approach that involves a random forest-based machine learning algorithm for SoM prediction, and a literature-derived database of biotransformation rules encoded using SMIRKS notation (de Bruyn Kops et al., 2019; de Bruyn Kops et al., 2021).

#### 2.1.2 Machine Learning-Based Pre-Experimental Metabolite Generation

Machine learning is a computational strategy that builds a prediction algorithm based on existing knowledge. The model processes training data to find patterns, which are captured in the algorithm. When the initial training is complete, the algorithm may be refined to consider new data for metabolite prediction (Finkelmann et al., 2018; Göller et al., 2020). This update process may be automated, but even in an unsupervised mode it may require significant time for statistical analysis.

Machine learning models require atom representations that capture reactivity-determining features of a potential reaction site (Rydberg et al., 2010; Matlock et al., 2015). This computational strategy is differentiated from rule-based models in several ways. Machine learning models are not limited by pre-determined rules, allowing them to consider a broader range of metabolic pathways. Deploying prediction software based on machine learning often requires data from the previous experimental MetID studies, which takes resources to collect and manage.

Most machine learning software does not organize its computational logic into human-readable rules, meaning interpretation is challenging (Kirchmair et al., 2015; Kazmi et al., 2019). This is relevant to MetID researchers, as metabolite predictions may be involved in research decisions and regulatory filings.

Examples of MetID software using machine learning models include

• XenoSite server: Provides tools for visualizing the atom most likely to be the site of metabolism for several important cytochrome P450s (CYP450s). XenoSite server uses a neural network machine learning model (Matlock et al., 2015).

• MetScore: Uses a random forest-based approach for predicting Phase I and II metabolism. Employs a quantum-chemistry derived molecular representation for reactivity prediction (Finkelmann et al., 2017).

• SMARTCyp: Employs a ligand-based CYP450 SoM prediction method with precalculated quantum mechanical activation energies to estimate site reactivity. Predictions are adjusted based on site accessibility (Rydberg et al., 2010; Olsen et al., 2019).

#### 2.1.3 Docking-Based Pre-Experimental Metabolite Generation

Docking-based approaches for pre-experimental computational strategies use 3D structural information about drug molecules to predict how they interact with CYP450s. Docking can be performed with parent compound, in which case the best-fitting pose would indicate the preferred SoM (Li et al., 2011). Alternatively, metabolite structures could be docked using a hybrid docking/rule-based approach (Tarcsay et al., 2010):

1. Rule-based generation of metabolites
2. Docking metabolites in CYP450 reaction sites
3. Selection of probable metabolites based on complementarity

The added structural context potentially increases the accuracy of docking-based prediction models, though this information may not be available. Docking-based models are typically limited to CYP450 and do not cover other activity from the human liver microsome (HLM).

Most research into docking-based metabolite prediction does not use a single software package. They instead employ a combination of tools to complete the analysis (Tarcsay et al., 2010; Moors et al., 2011). This offers flexibility but may be an obstacle to user experience and productivity.

Examples of docking-based software include

• IDSite: Evaluates the energy of a protein-ligand complex and employs a docking tool (GLIDE) to place the ligand into the active site. This is combined with a structure modeling program (PLOP) to determine binding orientations and predict SoM (Li et al., 2011).

• MetaSite: Employs an approach sometimes referred as pseudo-docking (Tyzask and Kirchmair, 2019). This software predicts potential SoMs by aligning ligand structures to GRID molecular interaction fields, which encode the active site “fingerprints” of cytochrome enzymes (Cruciani et al., 2005).

### 2.2 Post Experimental MetID Tools

Post-experimental MetID software uses predicted and experimental data to identify and verify metabolic products. The structure of a parent compound is used to predict metabolite structures, as described above. The application then assesses the analytical data to determine which of these theoretical chemicals are present.

Using a post-experimental prediction can significantly enhance the accuracy and reliability of a MetID study. This improved accuracy comes at the cost of requiring experimental data. Since a primary objective of pre-experimental MetID software is to avoid the need for unnecessary metabolism experiments, post-experimental predictive software should not be considered a substitute. Post-experimental predictive software is designed to accelerate metabolite data analysis and provide an added level of verification.

• Mass-MetaSite: Automatically identifies the metabolites for small molecules and peptides using liquid chromatography-mass spectrometry, UV, fluorescence, and radio-chromatogram data. Chemical structures are assigned to chromatographic peaks based on the MS and MS/MS fragmentation patterns (Trunzer et al., 2009).

• MZmine: mass spectrometry analytical data processing tool with metabolite identification capabilities (Pluskal et al., 2020). Metabolite structures are determined by compound database searches that may involve predictions by a machine learning algorithm (SIRIUS/CSI:FingerID) (Dührkop et al., 2019).

## 3 MetaSense Operation and Functionality

MetaSense^®^ is a metabolite prediction package developed by ACD/Labs which employs both rule-based and machine learning to perform pre-experimental and post-experimental predictions simplifying the review process for the expert. Understanding its functionality provides an instructive example of how MetID software operates.

### 3.1 Pre-Experimental Metabolite Generation

**FIGURE 1:**
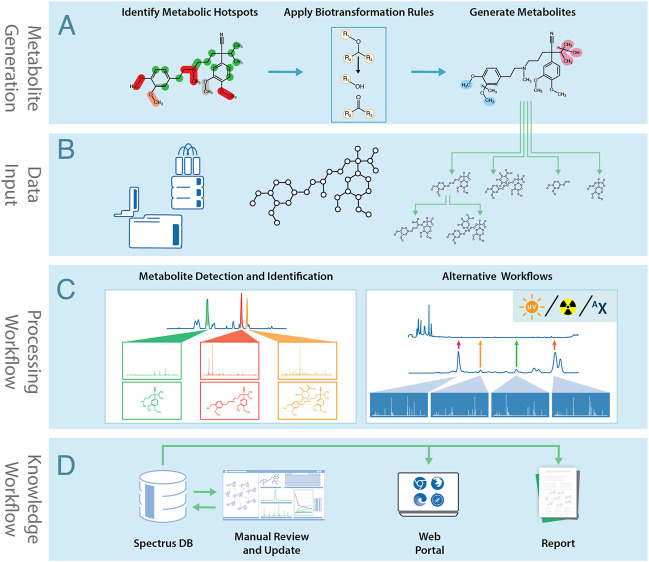
The MetaSense metabolite generation and identification process: **(A)** Metabolites are generated using structural information and biotransformation rules. **(B)** Data acquired by analytical instruments is combined with the molecular structure and biotransformation predictions. **(C)** Analytical data is processed to identify metabolites. **(D)** Processed data is stored in SpectrusDB database. Data can be manually reviewed and processed, accessed *via* software tools, or be used to generate reports.

MetaSense’s metabolite generation process is summarized in Figure 1A. The engine consists of two main components:

1. A database of biotransformation rules maps the chemical environment of a potential SoM to a list of expected reaction products. The biotransformation rule set was compiled from several review publications (Testa and Krämer, 2007a; Testa and Krämer, 2007b; Testa and Krämer, 2008; Dalvie et al., 2002) and further extended by analyzing the internal database of CYP450 substrates and their metabolites. The rules are grouped according to these reaction types:
  • Phase I—Hydrolysis: spontaneous or enzymatic hydrolytic cleavages of labile functional groups
  • Phase I—Redox reactions, including hydroxylations, dealkylations, heteroatom oxidations, epoxides formation, and ring desaturation with subsequent aromatization
  • Phase II—Conjugation reactions, such as sulfonation, glucuronidation, the addition of glutathione, and various amino acids
2. A soft spot ranking algorithm estimates the likelihood of metabolic reactions at a particular SoM. The scoring functions used depends on the reaction type.

Hydrolysis and Phase II stage scores are based on simple heuristics reflecting the overall lability of the SoM and susceptibility to conjugations based on the physicochemical profile of the parent compound. For example, the likelihood of lipophilicity reducing conjugations (e.g., sulfonation, glucuronidation) is assessed by predicted log*D*_7.4_ values, producing lower scores for inherently polar and water-soluble molecules. Conversely, glutathione addition is purely rule-based–its target sites are identified by substructure search against a set of electrophilic fragments.

For redox reactions of Phase I, MetaSense uses machine learning based regioselectivity prediction models that it shares with the ACD/Labs Percepta platform. These models identify soft spots for five common redox reaction types catalyzed by CYP450s and other metabolic enzymes expressed in the HLM.

The models use the GALAS (Global, Adjusted Locally According to Similarity) method, which can be described as a combination of two procedures:

• A fragmental baseline QSAR model for the prediction of the property of interest
• A similarity-based routine (or local model) that introduces additional corrections based on the analysis of the performance of the baseline model on the most similar compounds identified in the training set.

A specialized structural fragmentation method has been developed to account for the regioselectivity of enzymes on an atom-by-atom basis. Unlike traditional fragmentation techniques that use one “digital image” of the whole molecule, this regioselectivity model uses several unique molecule representations depending on the selected central atom. This fragmentation method and the weighing scheme for atoms surrounding the reaction center comprised the GALAS method variation for atom-centered predictions (Dapkunas et al., 2009).

The output of this model is the probability of a particular atom being a target of HLM enzymes, along with a reliability index—a quantitative measure of prediction confidence based on the local similarity correction step. The reliability index consistently predicts quality estimates by demonstrating a direct correlation between this value and accepted model accuracy metrics for both quantitative (Sazonovas et al., 2010) (e.g., MAE, RMSE) and qualitative (e.g., sensitivity, specificity) models (Dapkunas et al., 2009; Didziapetris et al., 2010). Finally, the two outputs from a GALAS HLM regioselectivity model are combined to produce the overall SoM score ranging from 0 to 1.

Once the SoM scores are calculated for all possible reaction sites in the molecule, they are filtered by a score threshold. Biotransformation rules are applied to generate a list of proposed metabolite structures. This process can be repeated to produce a biotransformation map (BTM). Products can be filtered by molecular weight, reaction type, or custom biotransformation rules.

### 3.2 Post-Experimental Metabolite Identification

The MetaSense post-experimental workflow is summarized in Figures 1B–D. The process starts by importing experimental data files and corresponding structures into the processing environment. This may include data from LC/MS/MS, radiotrace, UV-trace, or isotopically-enriched workflows. The software can use data from most major instrument vendors and metabolite prediction software, such as Meteor Nexus (Lhasa Ltd.), MetaSite (Molecular Discovery), or user-created SDFiles.

After processing, interpreted spectra are uploaded to a central database, and the BTM is automatically created. Scientists can review the entire project and add missing metabolites based on expert knowledge.

LC/MS traces are separated into extracted ion chromatograms (XIC). Predicted metabolites are matched to peaks by accurate mass and isotopic pattern. Since each XIC may contain peaks from several metabolites, the biotransformation site is located with MS/MS spectra by applying fragmentation rules and fragment-ion mass shifts. Metabolite structures are represented using Markush notation if the reaction site is ambiguous. The software also supports data-dependent acquisition, all-ion fragmentation, and MS^E^.

Unexpected metabolites are identified by control-sample comparison and fractional mass difference. Since all data and interpretations are linked and stored together, users can review original chromatograms and spectra and send them to other software tools for structure elucidation.

MetaSense offers two distinguishing features

• Auto-creation of BTMs, which are time-consuming to create manually. BTMs and kinetic plots are generated based on structural and experimental data. MetaSense uses chemical intelligence to refine BTMs, excluding chemically unfeasible steps.
• Storage of analytical and chemical data in a searchable database, including peak areas, metadata, maps, and plots. Analytical data can be reprocessed and updated if new metabolites are found.

### 3.3 MetaSense Terfenadine Example

**FIGURE 2:**
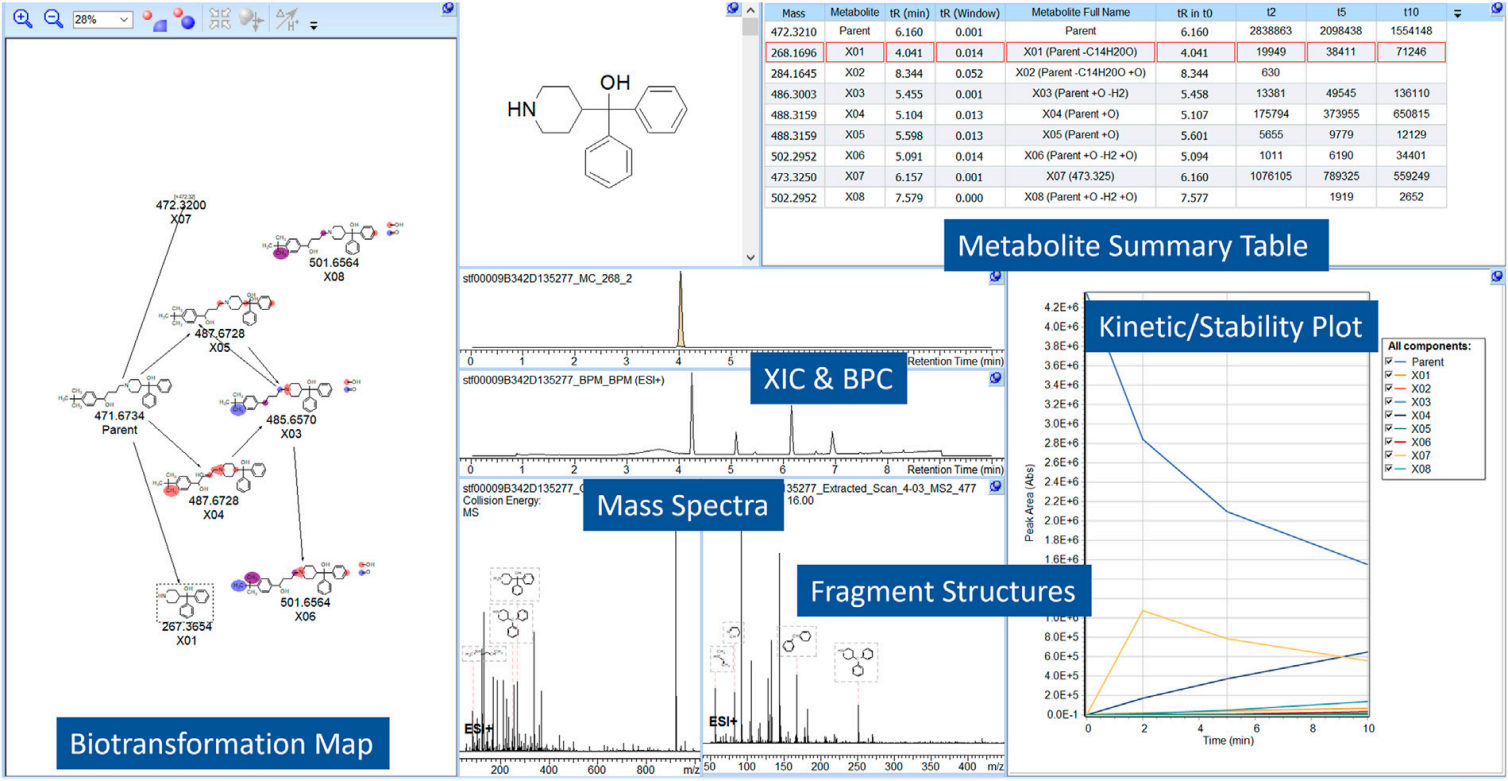
A screen capture from MetaSense, showing the analysis of Terfenadine. The reactions present, absolute area, retention time and mass are notated in the Metabolite Summary Table (i.e., Parent + O) and resultant structures are visualized in the BTM. The Kinetic/Stability Plot allows users to assess formation/generation of metabolites across the entire study.

A time-course metabolite study of Terfenadine is shown in Figure 2. Pre-experimental metabolism prediction reactions (Phase I/II) were set and filtered using post-experimental ID in the datasets. The feasibility of the structures was assessed through spectral assignment of predicted fragments. The areas of the parent or metabolites are visualized, allowing users to assess the formation of metabolites across the study.

## 4 Discussion

MetID scientists have access to more data than ever before. This includes experimental data from high-resolution instruments and in silico data produced by computer models. Effective data handling is a significant challenge. We predict that breakthroughs in productivity will be due to improvements in data management.

### 4.1 Interconnected Representations of Data

Data from metabolism studies are highly interconnected, but they can be organized into four layers: raw data, metadata, processed data, and interpretation.

• Raw data from an instrument detector is the foundation of metabolite data.
• Raw data is connected to metadata, including instrument conditions, chemical structures, model organisms, sample type, and sample preparation method.
• Raw data and metadata are then processed into ion traces and integrated peaks.
• Processed data is then abstracted into an interpretation layer, including BTMs, kinetic plots, and other visualizations.

MetID experts must understand the connections between these layers of data and interpretation. Automating the production of BTMs and kinetic plots from raw data saves time. Higher-level abstraction must be connected to raw data to support a rigorous analysis and identify chains of evidence for reporting and regulatory review.

These four layers of data and their connections should be accessible for review. Most MetID software does not meet this standard, as these tools do not allow hyperlinking between experimental design-processed data–raw data. Innovations in digital tools will overcome these restrictions, enabling researchers to track connections between multiple data layers.

### 4.2 Analytical Data Management Strategy

The productivity of MetID software is directly related to the quantity and quality of data available. This is ultimately determined by the data management strategy of a research organization. Therefore, analytical data management systems should be considered essential to any MetID program.

An analytical data management system must be designed to meet the needs of the overall research organization. This requires a balance between the functional needs of specific researchers and practical considerations such as expense, deployment time, or forward compatibility. Some of the most relevant concerns include:

• Data storage policy: While it is theoretically ideal to store every piece of data for all time, this is not practical or cost-effective. What features need to be stored to prepare for future data mining and machine learning?

• Findability: Findable data includes sufficient metadata to be readily retrieved. Lost data often requires experiments to be repeated, leading to increased time and cost. Findability can be improved with robust business practices surrounding metadata management.

• Homogenous file formatting: Data must be maintained in a long-term usable format. Data should be stored in a consistent, vendor-neutral format to reduce barriers to access, facilitate interoperability, and simplify data comparison.

• Current needs vs. future expansion: Local databases designed to meet the needs of a single laboratory can be deployed rapidly with minimal overhead. Decentralized systems such as this may not be conducive to machine learning projects or inter-laboratory collaboration. Enterprise systems require more effort to deploy and manage but can be designed to facilitate data science projects.

• Role of legacy data: Research organizations accumulate a massive volume of data. Legacy data may not have been processed or databased according to current best practices. Should this data be managed separately, or can it be used alongside new data? Does it require reformatting, reprocessing, or other forms of upkeep?

Each research function has specific database features they prioritize. MetID scientists benefit from searchable databases that include BTMs and summary tables.

## 5 Looking Forward

The accuracy and efficiency of metabolic studies have significantly increased due to improvements in instrumentation and software. The next challenge will be scaling processing and prediction tools to manage the volume of data generated by modern analytical equipment.

Applications should be developed to represent connections between raw data, metadata, processed data, and interpretation. We need dedicated tools to understand the relationships between these four data layers.

Machine interpretation relies on efficiently marshalling and curating data. Research organizations need to invest in systems that support successful MetID computer models. Innovations in drug metabolism prediction and identification will guide scientists to develop safer, more effective medication.

## Data Availability

The original contributions presented in the study are included in the article, further inquiries can be directed to the corresponding author.
